# Waterborne toxoplasmosis investigated and analysed under hydrogeological assessment: new data and perspectives for further research

**DOI:** 10.1590/0074-02760150262

**Published:** 2015-11

**Authors:** Flávia Pereira Vieira, Maria da Glória Alves, Livia Mattos Martins, Alba Lucínia Peixoto Rangel, Jitender Prakash Dubey, Dolores Hill, Lilian Maria Garcia Bahia-Oliveira/

**Affiliations:** 1 Universidade Estadual do Norte Fluminense Darcy Ribeiro, Centro de Biociências e Biotecnologia, Laboratório de Biologia do Reconhecer, Campos dos Goytacazes, RJ, Brasil; 2 Universidade Estadual do Norte Fluminense Darcy Ribeiro, Centro de Ciência e Tecnologia, Laboratório de Engenharia Civil, Campos dos Goytacazes, RJ, Brasil; 3 United States Department of Agriculture, Beltsville Agricultural Research Center, Animal Parasitic Diseases Laboratory, Beltsville, MD, USA; 4 Universidade Federal do Rio de Janeiro, Laboratório de Imunoparasitologia, Macaé, RJ, Brasil

**Keywords:** *Toxoplasma gondii*, groundwater contamination, oocysts, hydrogeology, TgERP, geomedicine

## Abstract

We present a set of data on human and chicken *Toxoplasma gondii* seroprevalence that was investigated and analysed in light of groundwater vulnerability information in an area endemic for waterborne toxoplasmosis in Brazil. Hydrogeological assessment was undertaken to select sites for water collection from wells for *T. gondii* oocyst testing and for collecting blood from free-range chickens and humans for anti-*T. gondii* serologic testing. Serologic testing of human specimens was done using conventional commercial tests and a sporozoite-specific embryogenesis-related protein (TgERP), which is able to differentiate whether infection resulted from tissue cysts or oocysts. Water specimens were negative for the presence of viable *T. gondii* oocysts. However, seroprevalence in free-range chickens was significantly associated with vulnerability of groundwater to surface contamination (p < 0.0001; odds ratio: 4.73, 95% confidence interval: 2.18-10.2). Surprisingly, a high prevalence of antibodies against TgERP was detected in human specimens, suggesting the possibility of a continuous contamination of drinking water with *T. gondii* oocysts in this endemic setting. These findings and the new proposed approach to investigate and analyse endemic toxoplasmosis in light of groundwater vulnerability information associated with prevalence in humans estimated by oocyst antigens recognition have implications for the potential role of hydrogeological assessment in researching waterborne toxoplasmosis at a global scale.

*Toxoplasma gondii* is the causative agent of toxoplasmosis, one of the most common parasitic zoonoses in humans worldwide. The ingestion of tissue cysts in undercooked meat or of sporulated oocysts in contaminated food or water results in two modes of transmission in humans. In epidemiological terms, it is not possible to determine the predominant route of transmission (oocysts or tissue cysts) by conventional serology (Jones & Dubey 2010). The same routes (oocysts and tissue cysts) can infect warm-blooded animals, resulting in human exposure to infective meat-tissue cysts (Dubey et al. 2005). Water is recognised as a vehicle for disseminating *T. gondii* oocysts. However, the presence of *T. gondii* oocysts has been infrequently documented in water sources (de Moura et al. 2006) and the evidence for waterborne transmission has been inferred mainly from epidemiological parameters (Bowie et al. 1997, Bahia-Oliveira et al. 2003). Geological and/or geographic factors affecting the quality of water consumed by people who do not have access to treated water can provide insight into the high prevalence of waterborne diseases worldwide. Hence, knowledge of geographic and/or geological variables as well as other environmental parameters that affect the quality of water consumed by poor populations may be a starting point for the adequate adoption of public health policies to cope with this difficult problem.

Global efforts have been made to reduce risk factors for waterborne diseases worldwide. In this sense, the eighth phase of the International Hydrological Programme (IHP-VIII), namely, the eight-year medium-term strategy of United Nations Educational, Scientific and Cultural Organization (2014-2021) (unesco.org/new/en/natural-sciences/environment/water/ihp/about-ihp/), aims to improve water security in response to local, regional, and global challenges*via* multidisciplinary and environmentally healthy approaches to water resources management. The present study's use of hydrogeological assessment to improve our understanding of environmental data influencing waterborne toxoplasmosis is aligned with the IHP-VIII aims. We analysed human and chicken *T. gondii*serology in light of information on groundwater vulnerability using a previously established vulnerability map of unconfined aquifers from Campos dos Goytacazes, state of Rio de Janeiro, Brazil (Alves et al. 2009), an area of endemic waterborne toxoplasmosis (Bahia-Oliveira et al. 2003). The results show a clear and significant association between areas of higher groundwater vulnerability (areas with a higher probability of surface contamination) and the higher seroprevalence of *T. gondii* in free-range chickens. For humans, the use of an ELISA for a sporozoite-specific protein [*T. gondii* embryogenesis-related protein (TgERP)], which differentiates oocyst and tissue cyst-induced infections (Hill et al. 2011), showed that the oocyst infection route is of significant magnitude. We discuss the possibility of anti-TgERP antibodies as new potential tools to indicate recent exposure to *T. gondii* oocyst antigens and/or a continuous*Toxoplasma* re-infection phenomenon in endemic settings where water has been identified as a risk factor for human infections.

## SUBJECTS, MATERIALS AND METHODS

*Study area and the vulnerability map of unconfined aquifers* - Campos dos Goytacazes (21º45'15”S 41º19'28'W) is a city in the north of RJ. Campos dos Goytacazes can be classified as having a tropical wet-and-dry climate type and an average annual temperature between 20-23ºC, with a maximum average temperature of 32ºC. The average annual rainfall is 1,300 mm unevenly distributed, with dry periods of high temperatures (FEEMA 1993). This is the largest municipality in the state, with an area of 4,032 km^2^ and a population of 480,648 inhabitants (IBGE 2014).

**Fig. 1:**
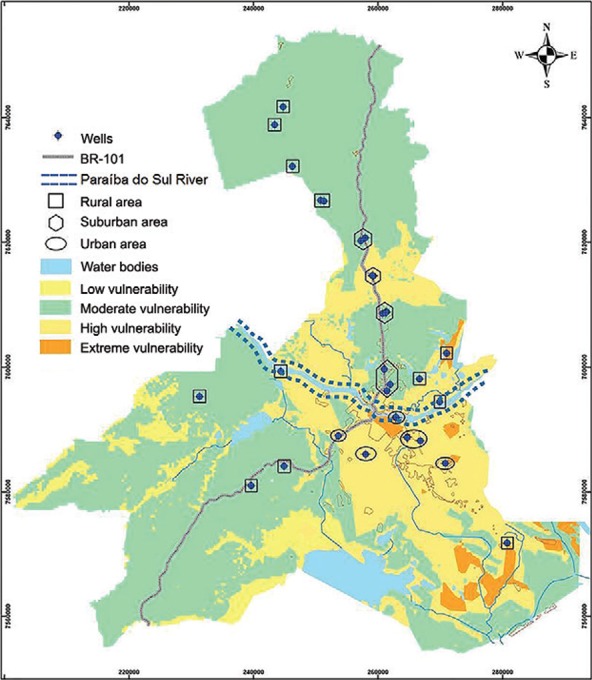
Campos dos Goytacazes, state of Rio de Janeiro, Brazil, groundwater vulnerability map generated according to DRASTIC methodology (Alves et al. 2009). The blue diamonds indicate the 40 household wells with a depth between 1-10 m. Urban (the central area of the map outlined by the ellipse), suburban (outlined by open diamonds) and rural (outlined by squares) areas are indicated.

The present study was undertaken using a previously established vulnerability map of unconfined aquifers from Campos dos Goytacazes using DRASTIC methodology. The acronym DRASTIC stands for “depth of the aquifer, recharge, aquifer media, soil media, topography, impact of the vadose zone and hydraulic conductivity” (Alves et al. 2009). This map was used to select residential sites for sampling human and animals to investigate serology against*T. gondii*. A total of 64 sites in urban, suburban and rural areas were investigated. Wells were present on 90% (57 of 64) of the visited properties. Seven properties did not have wells; they were located in urban areas and received water from the two municipal water treatment plants (Bahia-Oliveira et al. 2003). Two categories of aquifers occur in the Campos dos Goytacazes region: fractured and unconfined porous aquifers. The latter, which are the focus of this study, are composed of sandy and clay-rich sediments of the Quaternary, sediments of the Barreiras Formation of the Tertiary and residual soils from weathering of Precambrian rocks (Alves et al. 2009). The risk of groundwater pollution depends on the characteristics of the aquifer (geological formation containing or conducting groundwater) and the subsurface contamination load, which is influenced by anthropic actions (Aller et al. 1987, Foster et al. 1987, Gogu & Dassargues 2000, Panagopoulos et al. 2005, Bojórquez-Tapia et al. 2009). The groundwater vulnerability map presented in Fig. 1 was generated using DRASTIC methodology, a frequently employed method that considers the relevance of weighted hydrogeological factors (Aller et al. 1987). Geoprocessing techniques and the software programs ARCVIEW 3.2 and ARCGIS 9.0 were used in the elaboration and crossing of the thematic maps, resulting in the four classes/categories of vulnerability shown in the map in Fig. 1. Four indices (classes/categories of vulnerability) were identified: from 23-119, from 120-149, from 150-179 and from 180-230 (Alves et al. 2009). For the present study, the levels of probability of contamination (levels of vulnerability) from soil pollution were the same as previously (Alves et al. 2009). The categorisation attributed to each index referent to the levels of vulnerability was low (23-119), moderate (120-149), high (150-179) or extreme (180-230) (Alves et al. 2009). In demographic terms, such areas presented urban, suburban or rural features, as shown in Fig. 1. Because of the scarcity of people and free-range chickens living in areas of low vulnerability, these areas were not included in the study.

*Study population* - A total of 133 individuals living in the 64 visited properties (64 households) were invited to participate in the study; 128 (96%) consented (own or their guardians') after informed about the project. The study protocol complied with the Declaration of Helsinki and was approved by the local ethical committee (Research Ethical Committee of the Oswaldo Cruz Foundation, RJ; ruling 347/06) and the Brazilian National Council of Ethics in Research, Ministry of Health, (ruling 013/2007). The individuals (n = 128) lived in houses located in areas of moderate (n = 53, aged from 8-79 years), high (n = 60, aged from 8-84 years) or extreme (n = 15, aged from 11-71 years) vulnerability, as shown in the Table. Five millilitres of blood was collected from each person for the *T. gondii *serological assays. Standard serological tests (VIDAS Toxo IgG and IgM Kit, BioMérieux^®^, and ARCHITECT Toxo IgG, and IgM Kit, Abbott Diagnostics Division) that detect previous*T. gondii* exposure without discriminating the route of infection were performed to evaluate acute and chronic *T. gondii*human infection.

**TABLE:** Human and chicken *Toxoplasma gondii* seroprevalence by degree of vulnerability of groundwater to contamination in an area endemic for waterborne toxoplasmosis in Brazil

Groundwater vulnerability	Chickens				Human							
	n (n = 197)	Total seropositive n (%)	p^*a*^	OR (CI)	n (n = 128)	Age range (years)	Conventional ELISA- positives n (%)	p^*a*^	OR (CI)	TgERP ELISA- positives n (%)	p^*a*^	OR (CI)
Moderate	79	22 (27.8)			53	8-79	39 (73.6)			24 (45.3)		
High	70	44 (62.9)	< 0.0001^*b*^	4.38 (2.12-8.75)	60	8-84	49 (81.7)	0.3017	1.60 (0.65-3.91)	31 (51.7)	0.4981	1.29 (0.62-2.71)
Extreme	48	31 (64.6)	< 0.0001^*b*^	4.73 (2.18-10.2)	15	11-71	13 (86.6)	0.4917	2.33 (0.47-11.67)	8 (53.3)	0.5813	1.38 (0.44-4.36)

*a*: Fisher's exact test; *b*: statistically significant; CI: confidence interval; OR: odds ratio; TgERP: *T. gondii* embryogenesis-related protein.

*Oocyst investigation in water samples* - The presence of viable*T. gondii *oocysts in groundwater samples from wells in the studied area was investigated using chicken bioassays (de Moura et al. 2006). DNA extraction from fluoropore membranes was also performed with the FastDNA extraction method (Qbiogene, USA), by using a procedure previously published (da Silva et al. 1999). Water samples were collected from 40 previously georeferenced household wells located in the 64 properties in areas of moderate, high or extreme groundwater vulnerability. A total of 1,750 L of water was collected over a four-month period (an average of 50 L of water per well). The water was filtered through fluoropore 3 μm membrane filters (Millipore Billerica, USA) that were then fed by gavage to specific pathogen-free (SPF) chickens for bioassay, as previously described (de Moura et al. 2006). Four groups of chickens (total 10) were studied based on the vulnerability of water filtered through the membranes that they were fed by gavage: distilled water - control group (2 chickens), moderate vulnerability (2), high degree of vulnerability areas (3) and extreme degree of vulnerability (3).

*T. gondii serology in free-range chickens* - Serum samples from 197 free-range chickens inhabiting peridomestic areas of the 64 investigated households were tested for anti-*T. gondii* antibodies. The chickens were distributed as follows: 79 in areas of moderate vulnerability, 70 in areas of high vulnerability and 48 in areas of extreme vulnerability. The sera were evaluated using a modified agglutination test (MAT), as described by Desmonts and Remington (1980) and Dubey and Desmonts (1987); a titre of 1:25 was considered indicative of *T. gondii* exposure. The animal ethics protocol was approved under institutional protocol CEUA # 97.

*ELISA against TgERP* - To investigate the transmission route, the sera of all 128 human participants were also analysed for reactivity against TgERP, with the aim of differentiating oocyst-acquired infections from those acquired through tissue cysts (Hill et al. 2011). Briefly, testing for reactivity to TgERP was performed by ELISA. TgERP (uncleaved with Factor Xa) was diluted to a concentration of 2 μg/mL in 0.1 M carbonate buffer, pH 9.6. ELISAs were carried out essentially as described by Gamble et al. (2000). Reference positive and negative controls were included on each plate. A positive cut-off was established as the mean value of optical density (OD) of seronegative samples plus three times the standard deviation of seronegative samples. Plates were read at 405 nm using a Vmax ELISA reader. The levels OD against TgERP were arbitrarily considered as higher and lower than 1,000 in order to estimate the degree of positivity among those individuals who tested positive against this antigen. The degree of positivity against TgERP was considered high for individuals presenting values of OD higher than 1,000 and low for individuals presenting values of OD lower than 1,000.

*Statistical analysis* - Statistical analyses were performed using a chi-square test for linear trends and Fisher's exact test was used with a 95% confidence interval (CI) (GraphPad Prism 6) to assess significant associations between the seroprevalence of human and chickens toxoplasmosis and the groundwater vulnerability degree. Odds ratios (ORs) with 95% CIs were calculated.

## RESULTS

*Oocyst investigation in water samples* - Water samples collected from the 40 wells investigated in this study (Fig. 1) totalled 1,750 L collected over a four month period from June-September of 2013 (average of 50 L of water per well). The samples were filtered through 17 fluoropore 3 μm membrane filters that were then given to 10 SPF chickens for bioassay. Three chickens (2 from high-vulnerability areas and 1 from an extreme-vulnerability area) seroconverted to *T. gondii* infection when tested by MAT. However, viable *T. gondii* could not be isolated from these three chickens or from the seronegative chickens fed with membranes. Attempts to amplify parasite DNA from fluoropore membranes also yielded negative results.

*Serological findings in free-range chickens* - Serum samples from the 197 free-range chickens inhabiting peridomestic areas were evaluated using the MAT. The chickens were distributed as follows: 79 in areas of moderate vulnerability, 70 in areas of high vulnerability and 48 in areas of extreme vulnerability (Table). A significant association between seroprevalence and groundwater vulnerability was observed. Chickens from areas of high and extreme groundwater vulnerability were respectively 4.38 and 4.75 times more likely to be *T. gondii* seropositive than those from areas of moderate groundwater vulnerability (OR: 4.38, 95% CI: 2.19-8.74) for high vulnerability areas and (OR: 4.72, 95% CI: 2.18-10.2) for extreme vulnerability areas (Table).

**Fig. 2A:**
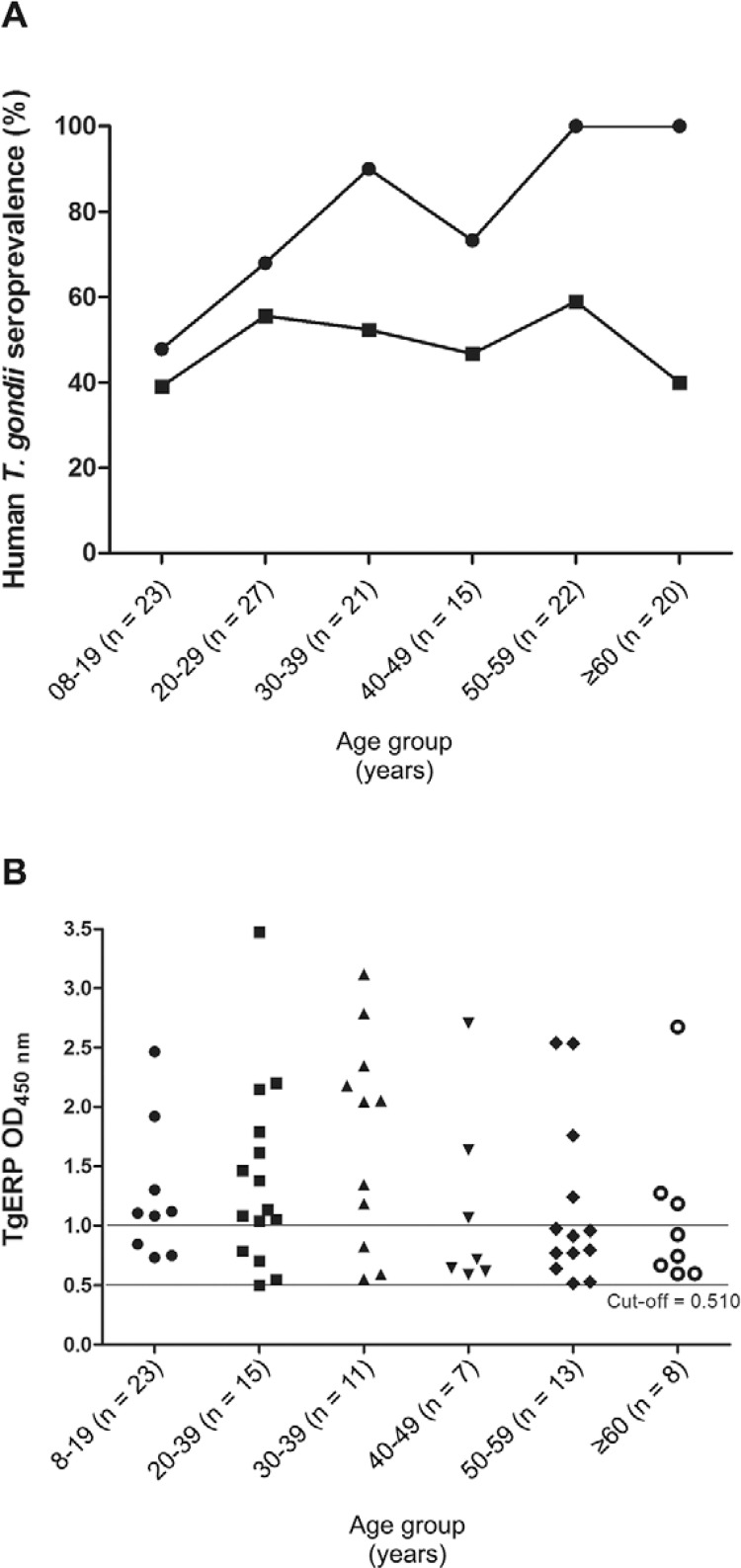
*Toxoplasma gondii* seroprevalence according to age, as determined by conventional ELISA (●) and *T. gondii*embryogenesis-related protein (TgERP) ELISA (■) for 128 individuals; B: degree of antigenic recognition, as assessed by TgERP ELISA according to the age range of the study population. Optical density (OD) value greater than 1,000 was established arbitrarily to regard individuals having higher degree of positivity against TgERP (see Subjects, Materials and Methods).

*Human seroprevalence of toxoplasmosis based on conventional and TgERP serology* - Serology in humans was evaluated by both conventional ELISA and TgERP ELISA, as shown in Fig. 2 and in theTable. In humans, the prevalence of IgG antibodies, as measured by both TgERP ELISA and conventional ELISA, was higher in areas with a higher degree of groundwater vulnerability (Table). However, no statistically significant differences in seroprevalence were observed when considering the degree of groundwater vulnerability. In Fig. 2A, the seroprevalence of conventional antigens and TgERP are compared as a function of the age range of the patients, independent of whether they were from areas of moderate, high or extreme groundwater vulnerability. The prevalence curves for conventional antigens and TgERP are parallel in the section of the curves where the slope is highest (from 8-29 years old); in this age range, six individuals were positive exclusively for TgERP (they were negative by conventional ELISA). After the age of 20, the seroprevalence of TgERP was stable at approximately 50%. None of these individuals had IgM levels compatible with recently acquired toxoplasmosis, as detected by conventional serology (data not shown). In Fig. 2B, the degree of antigenic recognition assessed by TgERP ELISA is expressed in terms of OD values higher or lower than 1,000 as a function of the age range of the groups, independent of whether patients were from areas of moderate, high or extreme groundwater vulnerability. Only TgERP-positive individuals (n = 63) are shown in Fig. 2B. The number of individuals presenting OD greater than 1,000 is higher for younger individuals groups and decreases with age, indicating that probably the infection is more recent in those individuals. The numbers in brackets represent the numbers of individuals in each age group in Fig. 2A, B.

## DISCUSSION

The DRASTIC methodology has been increasingly employed in Geographic Information System-based mapping to geospatially represent and predict groundwater quality and to provide the basis for policy decisions regarding protection of aquifers from chemical and biological pollutants. It has provided a useful method for evaluating long-term changes in the vulnerability of groundwater to microbiological and chemical pollution. Good agreement has been reported between the areas classified by this method as highly vulnerable and those that are found to have high levels of pollution based on field testing (Hammouri & El-Naqa 2008, Jamrah et al. 2008,Muhammad et al. 2015). Our application of DRASTIC methodology opens new perspectives in understanding environmental aspects of waterborne toxoplasmosis.

Transmission of *T. gondiivia* drinking water has been reported in the context of outbreaks and as a risk factor in toxoplasmosis-endemic areas (Jones & Dubey 2010, Karanis et al. 2013, Krueger et al. 2014). However, estimation of the levels of host exposure to*T. gondii* oocysts *via* drinking water is a complex and multifactorial subject. In Colombia, it was investigated the geographical factors influencing frequency of serological markers for congenital toxoplasmosis and it was found a significant correlation between mean rainfall at the city studied and the incidence of markers for congenital infection (Gómez-Marin et al. 2011).

Features including unknown levels of contamination from water sources and the fact that *T. gondii* can be transmitted by several modes contribute to a lack of specific recommendations to prevent the transmission of* T. gondiivia* drinking water (Dubey 2004).

We were not able to amplify DNA from the parasite or isolate the parasite despite the seroconversion of three chickens fed with membranes filtered water from wells classified as highly or extremely vulnerable, reinforcing the need to improve and/or to seek new alternatives to investigate (or estimate) the presence of *T. gondii* oocysts in water sources. In fact, the detection of viable*T. gondii* oocysts in sources of water is difficult because it depends on bioassays due to oocysts' inability to reproduce in vitro (Gilot-Fromont et al. 2012, Karanis et al. 2013). This is a disadvantage in comparison with other types of waterborne pathogens, such as those that multiply in vitro.

A direct and statistically significant relationship between higher groundwater vulnerability and higher *T. gondii *seroprevalence was observed only for chickens as far as we know it has not been previously described or investigated under this perspective. For human seroprevalence, this relationship was evident only as a tendency without statistical significance (Table). This difference between trends in chicken and human seroprevalence may be explained at least in part by the behaviour of humans with respect to their constant mobility across areas with diverse risk of *T. gondii *oocyst groundwater contamination, whereas chickens are confined to the same peridomestic areas during their lifespan and also by the differential habits and hygiene behaviour observed in humans. Regarding this last point, Casartelli-Alves et al. (2015) published a study on environmental contamination by *T. gondii* based on georeferencing isolates from chickens in an endemic area of RJ. The researchers applied questionnaires for risk factors that considered the ingestion of unfiltered and untreated water from natural sources, including wells and rivers. They found that in 100% of the 51 farms investigated, chickens drank untreated and unfiltered water. Humans used untreated water (unfiltered and not boiled) for drinking on 41.2% (n = 21) of the farms and treated water (filtered) for drinking on 58.8% (n = 30).

Free-range chickens are considered to be good indicators of soil contamination by*T. gondii *oocysts (Dubey 2010). The area studied had been previously described as having high levels of soil contamination with *T. gondii* oocysts (da Silva et al. 2003). Our observed strong association between positive *T. gondii* serology and higher degree of groundwater vulnerability reveals that chickens can be also good indicators for vulnerability of groundwater contamination with *T. gondii *oocysts and suggests that this further investigation is warranted.

We hypothesise that under hydrogeological conditions of groundwater vulnerability, if soils are (or have been, for a certain period) contaminated with *T. gondii* oocysts, as can be estimated based on free-range chickens' seroprevalence, there is a significant chance that the groundwater will be contaminated with *T. gondii* oocysts and will become an important and persistent source of parasite infection. Ground water would be a more favourable environment than soil for the persistence and “preservation” of oocysts and for the organism's evolution and perpetuation. In fact, soil contamination by *T. gondii* oocysts is not a stationary event because it can varies depending on the presence of feline excretion of oocysts in faeces and on environmental conditions such as temperature and moisture (Dubey 2004, Casartelli-Alves et al. 2015).

With this in mind, it is possible to consider the direct association observed between the degree of groundwater vulnerability and chicken seropositivity might represent a type of “vicious cycle” in which felines contaminate soil with *T. gondii* oocysts, which, by percolation, contaminate the groundwater or other water sources. Chickens and other birds can acquire *T. gondii*infection by drinking contaminated water and then, by carnivorism, felines are in turn infected. Corroborating this possibility there are at least two already reported aspects of environmental contamination. First, *T. gondii*oocysts remain viable in fresh and marine water for long periods (Lindsay et al. 2003). Second, free-range chickens (and birds in general) that drink unfiltered water from wells and other natural untreated sources of water (such as lakes or lagoons) can become infected (Casartelli-Alves et al. 2015). A third aspect that is consistent with this hypothesis is the hydrophilic nature and negative charge of *T.gondii *oocysts in freshwater (Shapiro et al. 2009), which could facilitate widespread contamination of groundwater from unconfined aquifers.

We did not detect a statistically significant association between human infections and vulnerability of aquifers. In a previous study in the same area, we showed that the magnitude of *T. gondii* transmission *via* oocyst ingestion is probably related to drinking water (Bahia-Oliveira et al. 2003). In our study, none of the individuals presented signs or symptoms of recent infections, and all were IgM negative, indicating the presence of antibody to TgERP in chronically infected individuals. However, we have only indirect evidences, our data on TgERP IgG levels in the individuals who, based on conventional serology, were chronically infected, indicates that (i) it potentially may be used in further studies to estimate of exposure to the *T. gondii* oocysts at the individual level and (ii) continual re-infection may be occurring in areas endemic for waterborne toxoplasmosis since after the age of 20, the seroprevalence of anti-TgERP was stable at approximately 50%.

The great genetic diversity of circulating parasites in Brazil, especially in Campos dos Goytacazes (Dubey et al. 2003, 2008, Su et al. 2012, Shwab et al. 2014), could contribute to the possibility of a gradual acquisition (with age) of immunity (a broader repertoire) against the plethora of antigenic diversity resulting from this genetic diversity. In fact, the *T. gondii* re-infection phenomenon has been well documented in the context of congenital toxoplasmosis and the basis of the evidence is the genetic diversity of the parasite possibly involved in the re-infection episodes documented (Elbez-Rubinstein et al. 2009).

Reinforcing this possibility is the fact that the six individuals who were negative by conventional serology and positive by TgERP ELISA in the present study were recently re-tested and continued to be negative by conventional serology (data not shown), however we cannot rule out the possibility of a cross-reactivity between TgERP antigens and antigens from other parasites for instance. Nonetheless, a viable explanation for these data is the possibility that the antigenic diversity of circulating parasites generates an immune response in the population that is not 100% covered by commercial tests against *T. gondii*; those tests are made with dominant antigens from parasite strains circulating in Europe and North America that present with lower genetic diversity in comparison with Brazilian strains. All of these data open avenues to investigate the possibility of the*T. gondii* re-infection phenomenon in endemic areas. In this sense, it will be necessary to investigate the recognition of antigens other than those used in conventional, commercially available serology as well as to investigate the presence of DNA parasite in peripheral blood as previously reported (Martino et al. 2005).

In conclusion, our data open new perspectives on experimental design and new avenues for understanding the dynamics of soil and groundwater contamination with *T. gondii*. For instance, controlled studies are warranted using hydrogeological assessment and field experiments with*Toxoplasma*-free sentinel chickens (Moré et al. 2012). Further, anti-TgERP IgG levels may be used to estimate frequency of individual *T. gondii* oocyst exposure and experimental studies are possible in which patients are followed using this serological parameter to evaluate how and the frequency with which it varies in controlled population-based studies.

Finally, the investigation of waterborne toxoplasmosis *via* this new approach may produce more consistent data using a lower number of human and animal serum samples in comparison with conventional investigations of *T. gondii*seroprevalence. These hydrogeological maps can provide a basis for planning strategies to reduce the risk of *T. gondii *infection in animals and humans. Furthermore, under the current circumstances of the world water crisis an urgent need has come into view for the agreement of coordinated and concerted actions involving interdisciplinary approaches to tackle with water safety questions. In this sense this novel presented approach may be replicated in other areas and also for other waterborne infections, not only in toxoplasmosis, what is possible because hydrogeological maps are increasingly being used to improve spatial and anthropic activities focused on preventing groundwater source contamination.

## References

[B1] Aller LT, Bennett T, Lehr JH, Petty RJ, Hackett G (1987). DRASTIC: a standardized system for evaluating ground water
pollution potential using hydrogeological settings.

[B2] Alves MG, Ramos IS, Coridola R (2009). Metodologia DRASTIC na análise de vulnerabilidade dos aquíferos
livres de Campos dos Goytacazes.

[B3] Bahia-Oliveira LM, Jones JL, Azevedo-Silva J, Alves CC, Orefice F, Addiss DG (2003). Highly endemic, waterborne toxoplasmosis in north Rio de
Janeiro state, Brazil. Emerg Infect Dis.

[B4] Bojórquez-Tapia LA, Cruz-Bello GM, Luna-González L, Juárez L, Ortiz-Pérez MA (2009). V-DRASTIC: using visualization to engage policymakers in
groundwater vulnerability assessment. J Hydrol.

[B5] Bowie WR, King AS, Werker DH, Isaac-Renton JL, Bell A, Eng SB, Marion SA (1997). Outbreak of toxoplasmosis associated with municipal
drinking water. The BC Toxoplasma Investigation Team. Lancet.

[B6] Casartelli-Alves L, Amendoeira MRR, Boechat VC, Ferreira LC, Carreira JCA, Nicolau JL, Trindade EPF, Peixoto JNB, Magalhães MAFM, Oliveira RVC, Schubach TMP, Menezes RC (2015). Mapping of the environmental contamination of Toxoplasma
gondii by georeferencing isolates from chickens in an endemic area in southeast
Rio de Janeiro state, Brazil. Geospat Health.

[B7] Silva AJ, Bornay-Llinares FJ, Moura IN, Slemenda SB, Tuttle JL, Pieniazek NJ (1999). Fast and reliable extraction of protozoan parasite DNA
from fecal specimens. Mol Diagn.

[B8] Silva DS, Bahia-Oliveira LM, Shen SK, Kwok OC, Lehman T, Dubey JP (2003). Prevalence of Toxoplasma gondii in chickens from an area
in southern Brazil highly endemic to humans. J Parasitol.

[B9] Moura L, Bahia-Oliveira LM, Wada MY, Jones JL, Tuboi SH, Carmo EH, Ramalho WM, Camargo NJ, Trevisan R, Graça RM, Silva AJ, Moura I, Dubey JP, Garrett DO (2006). Waterborne toxoplasmosis, Brazil, from field to
gene. Emerg Infect Dis.

[B10] Desmonts G, Remington JS (1980). Direct test for diagnosis of Toxoplasma infection:
method for increasing sensitivity and specificity. J Clin Microbiol.

[B11] Dubey JP (2004). Toxoplasmosis - a waterborne zoonosis. Vet Parasitol.

[B12] Dubey JP (2010). Toxoplasma gondii infections in chickens (Gallus
domesticus): prevalence, clinical disease, diagnosis and public health
significance. Zoonoses Public Health.

[B13] Dubey JP, Desmonts G (1987). Serological responses of equids fed To- xoplasma gondii
oocysts. Equine Vet J.

[B14] Dubey JP, Graham DH, Silva DS, Lehmann T, Bahia-Oliveira LM (2003). Toxoplasma gondii isolates of free-ranging chickens from
Rio de Janeiro, Brazil: mouse mortality, genotype, and oocyst shedding by
cats. J Parasitol.

[B15] Dubey JP, Hill DE, Jones JL, Hightower AW, Kirkland E, Roberts JM, Marcet PL, Lehmann T, Vianna MC, Miska K, Sreekumar C, Kwok OC, Shen SK, Gamble HR (2005). Prevalence of viable Toxoplasma gondii in beef, chicken,
and pork from retail meat stores in the United States: risk assessment to
consumers. J Parasitol.

[B16] Dubey JP, Velmurugan GV, Chockalingam A, Pena HF, Oliveira LN, Leifer CA, Gennari SM, Bahia-Oliveira LM, Su C (2008). Genetic diversity of Toxoplasma gondii isolates from
chickens from Brazil. Vet Parasitol.

[B17] Elbez-Rubinstein A, Ajzenberg D, Dardé ML, Cohen R, Dumètre A, Yera H, Gondon E, Janaud JC, Thulliez P (2009). Congenital toxoplasmosis and reinfection during
pregnancy: case report, strain characterization, experimental model of
reinfection, and review. J Infect Dis.

[B18] FEEMA (1993). Perfil ambiental do município de Campos.

[B19] Foster S, Ventura M, Hirata R (1987). Groundwater pollution: an executive overview of the Latin
America-Caribean situation in relation to potable water-supply.

[B20] Gamble HR, Andrews CD, Dubey JP, Webert DW, Parmley SF (2000). Use of recombinant antigens for detection of Toxoplasma
gondii infection in swine. J Parasitol.

[B21] Gilot-Fromont E, Lélu M, Dardé M-L, Richomme C, Aubert D, Afonso E, Mercier A, Gotteland C, Villena I, Djaković OD (2012). The life cycle of Toxoplasma gondii in the natural
environment. Toxoplasmosis - recent advances.

[B22] Gogu RC, Dassargues A (2000). Current trends and future challenges in groundwater
vulnerability assessment using overlay and index methods. Environ Geol.

[B23] Gómez-Marin JE, de-la-Torre A, Angel-Muller E, Rubio J, Arenas J, Osorio E, Nuñez L, Pinzon L, Méndez-Córdoba LC, Bustos A, de-la-Hoz I, Silva P, Beltran M, Chacon L, Marrugo M, Manjarres C, Baquero H, Lora F, Torres E, Zuluaga OE, Estrada M, Moscote L, Silva MT, Rivera R, Molina A, Najera S, Sanabria A, Ramírez ML, Alarcon C, Restrepo N, Falla A, Rodríguez T, Castaño G (2011). First Colombian multicentric newborn screening for
congenital toxoplasmosis. PLoS Negl Trop Dis.

[B24] Hammouri N, El-Naqa A (2008). GIS based hydrogeological vulnerability mapping of
groundwater resources in Jerash area - Jordan. Geofis Intl.

[B25] Hill D, Coss C, Dubey JP, Wroblewski K, Sautter M, Hosten T, Muñoz-Zanzi C, Mui E, Withers S, Boyer K, Hermes G, Coyne J, Jagdis F, Burnett A, McLeod P, Morton H, Robinson D, McLeod R (2011). Identification of a sporozoite-specific antigen from
Toxoplasma gondii. J Parasitol.

[B26] IBGE (2014). Diretoria de pesquisas, coordenação de população e indicadores
sociais.

[B27] Jamrah A, Al-Futaisi A, Rajmohan N, Al-Yaroubi S (2008). Assessment of groundwater vulnerability in the coastal
region of Oman using DRASTIC index method in GIS environment. Environ Monit.

[B28] Jones JL, Dubey JP (2010). Waterborne toxoplasmosis - Recent
developments. Exp Parasitol.

[B29] Karanis P, Aldeyarbi HM, Mirhasehemi ME, Khalil KM (2013). The impact of the waterborne transmission of Toxoplasma
gondii and analysis efforts for water detection: an overview and
update. Environ Sci Pollut Res Int.

[B30] Krueger WS, Hilborn ED, Converse RR, Wade TJ (2014). Drinking water source and human Toxoplasma gondii
infection in the United States: a cross-sectional analysis of NHANES
data. BMC Public Health.

[B31] Lindsay DS, Collins MV, Mitchell SM, Cole RA, Flick GJ, Wetch CN, Lindquist A, Dubey JP (2003). Sporulation and survival of Toxoplasma gondii oocysts in
seawater. J Eukaryot Microbiol.

[B32] Martino R, Bretagne S, Einsele H, Maertens J, Ullmann AJ, Parody R, Schumacher U, Pautas C, Theunissen K, Schindel C, Muñoz C, Margall N, Cordonnier C, Infectious Disease Working Party of the European Group for Blood and
Marrow Transplantation (2005). Early detection of Toxoplasma infection by molecular
monitoring of Toxoplasma gondii in peripheral blood samples after allogeneic stem
cell transplantation. Clin Infect Dis.

[B33] Moré G, Maksimov P, Pardini L, Herrmann DC, Bacigalupe D, Maksimov A, Basso W, Conraths FJ, Schares G, Venturini MC (2012). Toxoplasma gondii infection in sentinel and free-range
chickens from Argentina. Vet Parasitol.

[B34] Muhammad AM, Zhonghua T, Dawood AS, Earl B (2015). Evaluation of local groundwater vulnerability based on
DRASTIC index method in Lahore, Pakistan. Geofis Intl.

[B35] Panagopoulos GP, Antonakos AK, Lambrakis NJ (2005). Optimization of the DRASTIC method for groundwater
vulnerability assessment via the use of simple statistical methods and
GIS. Hydrogeol J.

[B36] Shapiro K, Largier J, Mazet JA, Bernt W, Ell JR, Melli AC, Conrad PA (2009). Surface properties of Toxoplasma gondii oocysts and
surrogate microspheres. Appl Environ Microbiol.

[B37] Shwab EK, Zhu XQ, Majumdar D, Pena HF, Gennari SM, Dubey JP, Su C (2014). Geographical patterns of Toxoplasma gondii genetic
diversity revealed by multilocus PCR-RFLP genotyping. Parasitology.

[B38] Su C, Khan A, Zhou P, Majumdar D, Ajzenberg D, Dardé ML, Zhu XQ, Ajioka JW, Rosenthal BM, Dubey JP, Sibley LD (2012). Globally diverse Toxoplasma gondii isolates comprise six
major clades originating from a small number of distinct ancestral
lineages. Proc Natl Acad Sci USA.

